# Comparative effectiveness of manual therapy and band exercises combined with high-intensity walking for pain, posture, and cardiorespiratory health in older adults: a randomised clinical trial

**DOI:** 10.3389/fmed.2025.1654670

**Published:** 2025-10-29

**Authors:** Anna Arnal-Gómez, Laura Fuentes-Aparicio, Elena Marqués-Sulé, Lucas Monzani, Rocío Cogollos-de-la-Peña, Dagmar Pavlu, Gemma Victoria Espí-López

**Affiliations:** ^1^Department of Physiotherapy, Faculty of Physiotherapy, University of Valencia, Valencia, Spain; ^2^Multispeciality Research Group (PTinMOTION), Department of Physiotherapy, University of Valencia, Valencia, Spain; ^3^Ivey Business School at Western University, London, ON, Canada; ^4^Faculty of Health Science, Universidad Europea de Valencia, Valencia, Spain; ^5^Faculty of Physical Education and Sport, Charles University, Prague, Czechia; ^6^Exercise Intervention for Health Research Group (EXINH-RG), Department of Physiotherapy, University of Valencia, Valencia, Spain

**Keywords:** older adults, manual therapy, elastic band, pain, posture, cardiorespiratory

## Abstract

**Background:**

Musculoskeletal pain and cardiovascular and pulmonary disorders affect the growing population of older adults. Manual therapy has proven to be effective, however, research using elastic bands is still scarce. The aim of this manuscript is to compare the effects of elastic band program training versus a manual therapy protocol, both combined with high-intensity walking, on age-related health parameters, such as pain, posture, and cardiorespiratory variables.

**Methods:**

A multicentre randomised clinical trial was conducted on community dwelling older adults with non-specific musculoskeletal pain. Participants were divided into two groups: (1) Manual therapy and high-intensity walking group (MTWG), who received self-assisted manual therapy followed by supervised high-intensity walking and (2) Elastic band and high-intensity walking group (EBWG) who performed resistance exercises with elastic bands and high-intensity walking. Musculoskeletal pressure pain threshold in trapezius muscles, back pain intensity, postural pattern (change in thorax position and chest wall expansion) and cardiorespiratory variables (heart rate and oxygen saturation, SpO_2_) were measured before the intervention (T0), after the 4-week programme (T1) and after the 4-week follow-up (T2).

**Results:**

A total of 102 older adults (49 in the MTWG and 53 in EBWG) completed the study and were analysed. Five RM-MANCOVA models were specified. The EBWG showed significantly higher PPT scores than the MTWG across all data points (*p* < 0.01); a between-within-subjects effect was found for VAS scores (*p* < 0.05) (Model A). Statistically significant within-subject differences were found for the left acromion-to-bed distance scores (*p* < 0.05) but not for the right-side scores (Model B). Similarly, significant between-subject differences for the upper chest wall were found (*p* < 0.05), but not for the lower chest wall. However, univariate tests revealed significant within-subject for both the upper and lower chest wall scores. Finally, no significant differences were found in pulmonary function scores (Model D), heart rate (HR) or oxygen saturation scores (Model E).

**Conclusion:**

Both protocols reduce pain. Manual therapy helps with non-specific back pain and improves acromion-to-table posture, while elastic band treatment enhances trapezius pressure threshold and upper thoracic expansion. Neither improved lower thoracic mobility or respiratory variables.

## Introduction

Nowadays, life expectancy has increased, leading to an increase in the proportion of older adults worldwide, and it is estimated that between 2020 and 2030, the percentage of people over 60 years of age will increase by 34% (1). The aging process entails a series of changes at the physical, social and psychological levels, as well as in the regulatory systems of the body, mainly the cardiovascular, pulmonary and musculoskeletal systems (2).

Non-specific neck and low back pain are the leading causes of global disability (3), the most prevalent pain in older adults affecting the back and spine (4). Despite this, pain tends to be underestimated in this population (5, 6), although this does not imply that they should be content to live with pain. Treatment and relief options that improve older adults’ quality of life are required, as well as exploring pain management solutions. In this regard, at the musculoskeletal level, aging is associated with a decrease in muscle mass and strength, reduced mobility and physical functionality, also affecting the quality of gait and the ability to walk safely and functionally (7). As a consequence of the decrease in muscle strength, older adults generate unconscious adaptations to balance their body weight through adjustments in the spine, which ends up significantly affecting body posture, and at the same time can generate pain in the musculoskeletal system (8). Thus, previous studies related to musculoskeletal pain in older adults conclude that pain likewise increases with age (9).

On the other hand, postural pattern alterations are one of the changes that most affect older adults, and the cause seems to be multifactorial. Such adjustments derive mainly from alterations in the passive and active stabilizing elements of the spine (10). In this regard, the trapezius muscle has been identified to be closely related to the postural changes caused by age and to the respiratory pattern (11); accordingly, assessing this muscle in terms of pain perception may be of interest.

At the cardiovascular level, aging is associated with an increase in blood pressure, changes in the endothelial muscles, such as atherosclerosis, or a decrease in the ejection fraction and cardiac output, which can result in a greater number of thrombotic processes (12). During the aging process, the respiratory system also undergoes a decrease in the distensibility of the thoracic and pulmonary wall, decreased vital capacity and a decline in cough strength or mucociliary clearance, with an increased risk of infections and broncho-aspirations (13). Moreover, musculoskeletal changes in the thorax lead to a more kyphotic posture, restricted mobility of the rib cage and thus a tendency toward a restrictive pattern (14). The increase in thoracic hyperkyphosis induces a shift of the centre of gravity which may increase the number of falls (15) and, consequently, the risk of fractures (16).

To address these problems, physiotherapy can contribute as a suitable therapeutic option. First, physical activity is recommended in order to achieve healthy aging. According to the World Health Organization, older adults should perform at least 2 days of muscle-strengthening activity and at least 150 min of moderate-intensity or 75 min of high-intensity physical activity (17). Previous studies have shown the beneficial cardiovascular and musculoskeletal effects of aerobic exercise in the population (18), as well as improving quality of life and mood (19). The simplest and most economical form of aerobic exercise is walking. There are authors who point out the effects of walking on the physical, cognitive, and nociceptive levels in older adults specifically, highlighting aerobic exercise as one of the best ways to work on chronic musculoskeletal pain (20).

On the other hand, self-assisted manual therapy (MT) has been shown to be effective for various musculoskeletal conditions such a musculoskeletal pain, functionality, and posture in older adults (21). It is also important to promote a person-centered approach to care (22). Moreover, it has been observed that in older adults, treatment focused on the rib cage has positive results on respiratory parameters (23).

Other studies infer the importance of combining therapeutic muscle resistance and aerobic exercise to improve mobility, muscle quality and strength in the elderly (24–27). A number of studies suggest that resistance exercises with elastic bands seem to be beneficial for the relief of chronic musculoskeletal pain in the elderly, but the literature is scarce (28, 29). In this sense, previous studies carried out in older adults have shown efficacy of 8- to 10-week treatment programmes for improving muscle quality, physical performance (30), grip strength and cardiovascular parameters (31), as well as walking speed (32). However, these studies did not focus on parameters such as perceived pain intensity, posture pattern, or they are not easily reproducible by clinicians or patients because they lack detail in their application. Therefore, the use of resistance bands emerges as an option to improve musculoskeletal health, in terms of pain parameters, thoracic expansion, posture and possibly even cardiovascular or respiratory parameters.

Finally, it should be noted that multicomponent training strategies, including different capacities and aspects in a single session, promote an improvement in the general health condition of older adults; this is why they are currently described as a powerful tool for addressing functional disorders in this population elderly people (33). In addition, this type of training is typically carried out in groups, thus favoring socialization among participants (33). On the other hand, recent clinical guidelines also propose multimodal treatment as an effective strategy for the management of patients with chronic neck pain (34). It is also important to highlight that guided exercise, performed independently and supervised by professionals, is an effective strategy for older people to achieve the levels of exercise recommended by international guidelines (35).

In short, the benefits of self-assisted MT for musculoskeletal pain, functionality, and posture have been well established. We are also aware of the benefits of resistance band-based strength training for each variable, but we have yet to learn the differences between these variables, or whether one treatment is better than another or if they are similar. For all of the above, the hypothesis was that resistance elastic band exercises program can be as effective as MT both combined with high-intensity walking, in relation to musculoskeletal pain and posture, chest wall expansion, and cardiorespiratory variables.

The main aim of this study was to analyse and compare the effects of two physiotherapy programmes, elastic band training versus MT, both combined with high-intensity walking, on the improvement of age-related health parameters, such as pain, posture, chest wall expansion and cardiorespiratory variables.

## Methods

### Study design

A multicentre randomised clinical trial was conducted from October 2021 to May 2022 in the Faculty of Physiotherapy, University of Valencia (Spain) and the Faculty of Physical Education and Sport, Charles University (Prague). The potential bias arising from a sample of two culturally and socially different locations was controlled by the stay of some co-authors in the collaborating country. This exchange facilitated the transfer of the methodology for both the evaluation and execution of techniques uniformly. Additionally, monthly follow-up checks were conducted to monitor the consistency and accuracy of the procedure. Participants were divided into two groups: (a) MT and high-intensity walking exercise group (MTWG), who performed self-assisted MT followed by supervised high-intensity walking; (b) elastic band and high-intensity walking exercise group (EBWG) who received resistance exercise with elastic band plus supervised walking at high intensity. In both groups the intervention protocols were implemented as a group and were always guided by a physiotherapist. Both therapeutic interventions had a total duration of eight sessions, 2 days a week, for a period of 4 weeks. Study variables were measured at baseline (T0), after the 4-week intervention (T1), and at 4-weeks follow-up after the end of the intervention (T2); participants were asked not to replicate the interventions during follow-up.

The study was conducted following the guidelines of the Consolidated Standards of Reporting Trials (CONSORT) (36), and informed consent was obtained from all participants. Participants featuring the intervention protocol footage of this manuscript gave consent for open access publication. The study was approved by the Ethics Committee for Research on Humans of the University of Valencia (No. 1393203), and registered, as part of a larger study, in the ClinicalTrials.gov database (Identifier: NCT04345211).

### Participants

The present study included community-dwelling older adults who met the following inclusion criteria: aged 60–80 years, with non-specific (specific causes were ruled out after gathering background information) (37) musculoskeletal or osteoarticular chronic pain (>3 months) and non-smokers (>6 months). The exclusion criteria were inability to walk without technical aids or assistance, history of respiratory or cardiac condition, acute musculoskeletal or osteoarticular pain at the time of assessment, contraindications for manual therapy or exercise, and cognitive impairment (Mini-Mental State Examination <25 points) (38).

### Randomization and blinding

Participants were randomly assigned to the MTWG or the EBWG. Randomization was performed using Microsoft Excel by an external researcher. An independent blinded researcher who was unaware of participant allocation and of the aim of the study performed the assessments and recorded the data. A statistician conducted the analysis and interpretation of results.

### Outcomes

Before the session, procedures were explained in detail to all participants. Sociodemographic and clinical data were then registered, followed by the measurement of the study variables. The sociodemographic and clinical characteristics included age, sex, educational level, marital status, body weight (using a Tanita BC 601, TANITA Ltd., Tokyo, Japan), height (measured with a SECA 213 stadiometer, Seca Ltd., Hamburg, Germany), body mass index [BMI, (kg/m^2^)], and the Charlson’s Comorbidity Index (39) which scores 0 to 1 (no comorbidity), 2 (low comorbidity), and 3 to 10 (high comorbidity). A physiotherapist with 15 years of experience in treating musculoskeletal disorders in older adults conducted individual, face-to-face assessments.

#### Primary outcome

##### Musculoskeletal pain

*Pain pressure threshold (PPT)* was evaluated in trapezius muscles by the minimal pressure (kg/cm^2^) which induces pain by pressure algometry (Wagner Instruments FDK 20, Greenwich, United States of America) since the trapezius is one of the muscles where pain in older adults is most prevalent (4). It was assessed in the middle part of the anterior border of the upper trapezius, three times, bilaterally, with 30-seconds rest between them and taking the mean value for analysis. It has shown to be reliable ICC = 0.91 (IC del 95%: 0.82; 0.97) (40).

*Pain intensity*. Global non-specific perceived intensity of back pain in the last week was assessed by the visual analogue scale (VAS). The VAS ranges from 0 “no pain” to 10 being the “worst pain imaginable,” in relation to pain intensity “in the last 24 hours.” The VAS has a high internal consistency (0.92) (41).

#### Secondary outcomes

##### Postural pattern

*Thorax position* was assessed by measuring the distance (mm) between acromion and bed using a rigid standard plastic transparent ruler. Participants are requested to lie supine on a standard treatment table and adopt their natural relaxed posture, hands rested gently on the abdomen, arms placed by their sides, and the elbows flexed and resting against the lateral wall of the abdomen, placing the glenohumeral joint in slight internal rotation (42). The linear distance from the treatment table to the posterior (lateral) aspect of the acromion is then measured without exerting any downward pressure onto the table. Each side is measured three times in succession, and on each occasion the right angle was replaced as previously described. This test has shown to be reliable with an ICC between 0.92 and 0.93 for subjects with symptoms and between 0.90 and 0.93 for subjects without symptoms (43).

*Chest wall expansion*. A measuring tape is used to measure chest expansion in centimeters (cm) at two levels of the rib cage. For upper chest expansion, the anatomical landmarks used are the spinous process of fifth thoracic vertebrae, the midclavicular line, and the third intercostal space. For lower chest expansion, the anatomical landmarks used between the chest circumference at maximal inspiration and maximal expiration was measured with a tape measure. The measurements were repeated twice and the means of the two values obtained was considered. The difference between inspiration and expiration at each site was used for analysis. A lower value represents limited chest wall expansion (44).

##### Cardiorespiratory parameters

*Pulmonary function* was measured with pulmonary function tests. Spirometry was conducted with the Spirovit SP-10 (Contec, Barcelona) spirometer. Forced vital capacity (FVC), forced expired volume at 1 s (FEV1) and the FEV1/FVC ratio (%) by forced expiratory flow manoeuvre were obtained, and the FEV1/FVC ratio was used for analysis. The predicted percentage was registered and 80% or over of the reference value was considered as an optimal value (45); for the FEV1/FVC ratio, a decreased percentage allowed defining the concept of “obstruction” (46). The test was performed according to the American Thoracic Society standards (47). Spirometry has shown an intraclass correlation coefficient (ICC) of 0.89 in COPD patients (48).

*Heart rate (HR) and oxygen saturation (SpO_2_)* were measured using a finger pulse oximeter (Finger Pulse Oximeter FS20C with OLED display). Participants were seated and relaxed while the pulse oximeter was positioned on their index finger, ensuring it was clean and devoid of nail polish. After a few seconds for the pulse oximeter to detect the parameters, the HR and SpO_2_ were registered. The normal resting HR of healthy older adults is on average 80 bpm (range: 78–82 bpm) (49). The normative SpO_2_ levels at sea level are between 96 and 100% (50).

### Intervention

Two treatments guided by a physiotherapist with more than 20 years of experience in applying manual therapy were applied.

#### Self-administered manual therapy protocol

A self-administered manual therapy protocol was implemented, comprising a total of nine techniques based on a previous study (21) which included prior respiratory awareness of the abdominal and thoracic areas. Seven techniques focused on the cervical, scapular, and thoracic regions, and concluded with abdominal and thoracic respiratory awareness exercises. The whole protocol lasted 30 min.

#### Self-administered elastic band exercises protocol

Exercises were aimed at the upper limbs, torso, and lower limbs. Upper limb and torso exercises focus on body straightening and “chest opening” while lower limb exercises aims to improve the position of the pelvis, thus also the position of the torso and indirectly the chest. The upper limbs were not overburdened during the exercises (51). Each exercise was performed five times in a row, slowly executing each exercise and always returning to the starting position gradually resisting against band pull (Appendix I). Participants were asked to reach a rating of 16 (“somewhat hard”) on the Rate of Perceived Exertion (RPE) scale (52). The external band resistance was increased by adding more bands or by using bands with higher resistance through greater stretching. The intervention used green and red Akrafit bands,^1^ with the green and red bands providing between 13–20 N and 18–27 N of resistance, respectively, when stretched to twice their original length.

#### High-intensity walking exercise

Walking was performed immediately after MT or elastic band intervention, since exercise in this order has been shown to enhance the synergistic effect of combining the two interventions (53, 54). This protocol consisted of supervised walking on a 400 m circular track/terrain. In order to determine the exercise intensity when performing the walking protocol, the RPE scale (52) was used. The structure of the walking sessions began with a warm-up including a short walk at self-selected speed, with a duration of 10 min. After the warm-up, participants walked at a target intensity of 16 (“somewhat hard”) on the RPE scale for 40 min according to the same criteria as those used by Espí-López et al. (21) in their study.

### Sample size calculation

The sample size was calculated using G*Power 3.1.9.7. An “*a priori*” power analysis was performed for two independent groups to detect small to moderate effect sizes (Cohen’s *d* = 0.25, based on prior study) (55) with *α* = 0.05 and 1-*β* = 0.95. The results indicated a minimum sample size of 44 participants to achieve sufficient statistical power and we considered a loss of 10%, the sample needed for our study being in total 48 participants.

### Statistical analysis

All statistical analyses were conducted with IBM SPSS Statistics 29. First, Pearson’s bivariate correlations were calculated to determine any association between outcomes. A statistically significant correlation between outcomes would justify conducting repeated measures, multivariate analysis of variance, or RM-MANOVA. Box’s M and Mauchly’s Sphericity tests were conducted to ensure that our RM-MANOVA models met the assumptions of general linear models. A non-significant *p*-value in each of these tests would indicate that the homogeneity of variance assumptions (between-subjects) and the sphericity assumption (within-subjects) were met. See Appendix II for a more detailed explanation of our analysis and robustness checks.

Five RM-MANCOVA models were generated to test our hypotheses, mainly, Models A, B, C, D, and E. In all models, the dependent variable was measured at three data points, mainly (T0) baseline, (T1) post-intervention, and (T2) follow-up (1 month after; within-subjects factor). For our between-subjects factor, we entered a dummy coded variable, in which the control group was coded as “0,” and the treatment group was coded as “1.” No control variables were entered in any of the five models.

Model A tested for significant differences in our primary outcome, PPT, and overall VAS, Hypothesis 1a and 1b, respectively. Regarding our secondary outcomes, Model B tested for significant differences in participants’ thorax position measured as the distance from the posterior edge of the acromion to the flat surface (Hypothesis 2a). Similarly, Model C tested for differences in upper and lower chest wall expansion (Hypothesis 2b). Finally, models D and model E tested for significant differences in pulmonary function (Hypothesis 3a), and for differences in cardiovascular frequency and oxygen saturation (Hypothesis 3b).

## Results

**Figure 1:**
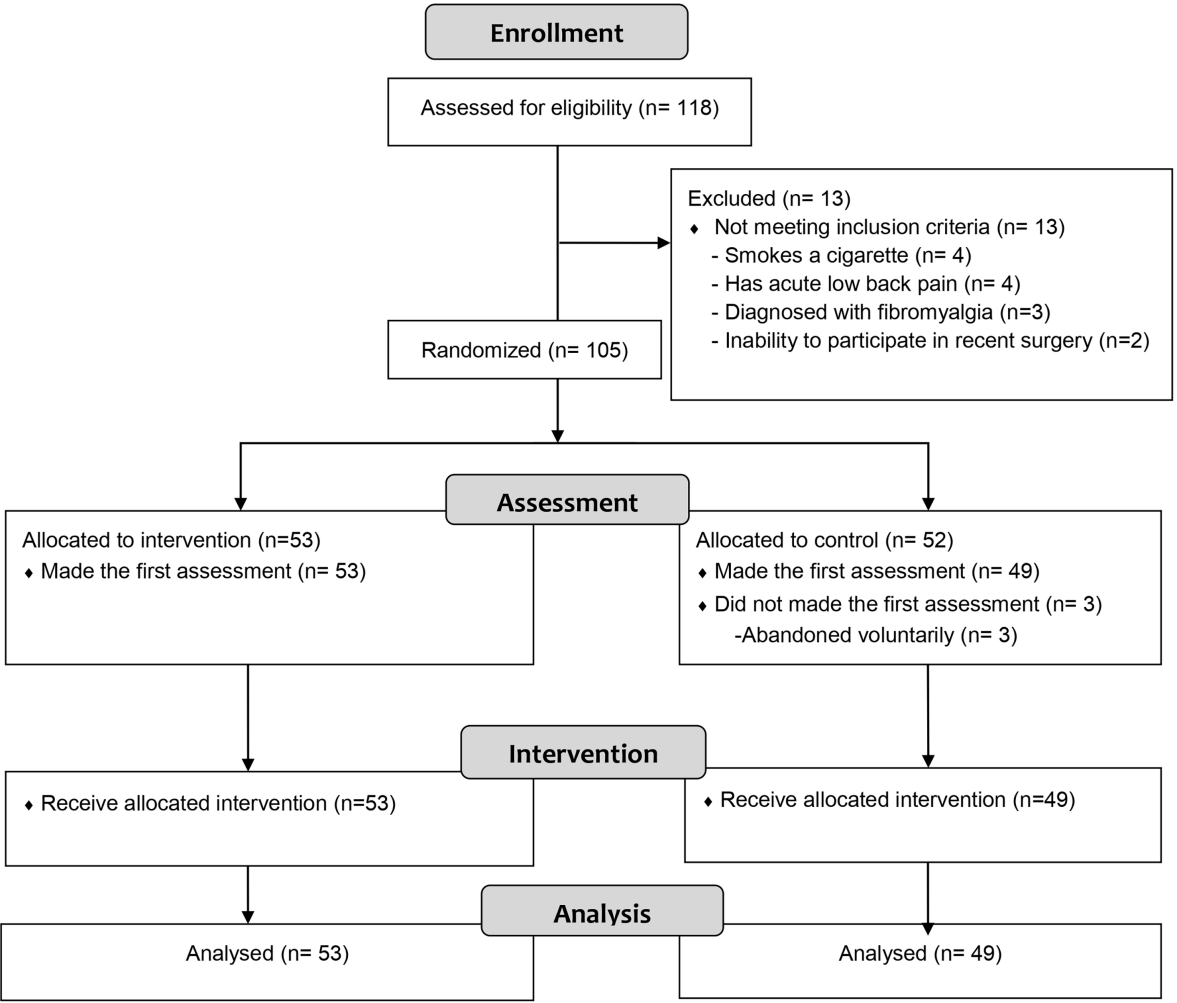
Flowchart according to CONSORT statement for the report of randomized trial.

Of the 118 initial participants, 105 met the inclusion criteria and were allocated to intervention. Before the intervention, some participants dropped out so ultimately, 102 (49 in the MTWG and 53 in EBWG) completed the study and were analysed (Figure 1). Table 1 shows sociodemographic data.

**Table 1:** Sociodemographic and anthropometric characteristics of the participants.

Outcomes	Categories/units	Total sample	EBWG	MTWG
		*n* = 102	*n* = 53	*n* = 49
Sex *n* (%)	Women	81 (79.40%)	45 (84.90%)	36 (73.50%)
	Men	21 (20.60%)	8 (15.10%)	13 (26.50%)
Age (IRQ)	Years	70 (6.50)	70 (6.00)	70 (7.00)
Marital status *n* (%)	Single	12 (11.80%)	7 (13.20%)	5 (10.20%)
	Married	55 (53.90%)	27 (50.90%)	28 (57.10%)
	Widowed	20 (19.6%0)	11(20.80%)	9 (18.40%)
	Divorced	13 (12.70%)	7 (13.20%)	6 (12.20%)
	Separated	1 (1%)	1 (1.90%)	–
	Other	1 (1%)	–	6 (12.20%)
Level of education *n* (%)	Primary	1 (0.90%)	–	–
	High School	43 (42.10%)	25 (47.20%)	20 (40.80%)
	University	58 (56.90%)	27 (50.90%)	29 (59.20%)
	No studies	1 (1%)	1 (1.90%)	
Employment status *n* (%)	Employed	7 (6.90%)	4 (7.50%)	3 (6.10%)
	Retired	95 (93.10%)	49 (92.50%)	46 (93.90%)
Weight (IRQ)	kg	67.70 (14.10)	67.70 (15.90)	67.40 (12.65)
Height (IRQ)	m	1.62 (0.12)	1.63 (0.12)	1.61 (0.12)
Body mass index (IRQ)	kg/m^2^	26.18 (4.24)	25.12 (5.26)	25.51 (4.58)
Medication *n* (%)	Yes	24 (23.5%)	14 (26.4%)	10 (20.4%)
Charlson comorbidity index (IRQ)		0.00 (0.00)	0.00 (0.00)	0.00 (0.50)
Hypertension *n* (%)	Yes	33 (32.40%)	19 (35.80%)	14 (28.60%)
High cholesterol *n* (%)	Yes	47 (46.11%)	26 (49.10%)	21 (42.92%)
Mini-mental cognitive test (IRQ)	Total scores	34 (2.00)	34 (3.00)	34 (2.00)
Smoking index (IRQ)		0.00 (0.00)	0.00 (0.00)	0.00 (14.50)

EBWG, elastic band and high-intensity walking group; MTWG, manual therapy and high-intensity walking group; IRQ, interquartile range.

Table 2 shows means, standard deviations and Pearson’s bivariate correlations. As expected, the three data points were correlated for our outcome measures. These correlations justify the use of an RM-MANOVA approach to isolate the effect of said correlation.

**Table 2:** Means, SD, and Pearson bivariate correlations.

Outcomes	M	SD	1.	2.	3.	4.	5.	6.	7.	8.	9.	10.	11.	12.	13.	14.
01. PPT-T0	3.45	1.50	–													
02. PPT-T1	3.90	1.80	0.72^**^	–												
03. PPT-T2	4.21	1.77	0.72^**^	0.79^**^	–											
04. VAS-T0	2.17	1.78	−0.20^*^	−0.15	−0.09	–										
05. VAS-T1	1.19	1.41	−0.16	−0.39^**^	−0.25^*^	0.46^**^	–									
06. VAS-T2	1.15	1.45	−0.17	−0.21	−0.17	0.61^**^	0.50^**^	–								
07. ACROM-R-T0	8.00	2.21	0.04	−0.01	−0.09	−0.22^*^	0.09	−0.14	–							
08. ACROM-R-T1	8.09	1.88	−0.01	0.06	−0.05	−0.20^*^	0.14	−0.07	0.78^**^	–						
09. ACROM-R-T2	7.81	1.92	0.11	0.11	0.04	−0.24^*^	0.06	−0.06	0.68^**^	0.75^**^	–					
10. ACROM-L-T0	7.74	2.07	−0.01	−0.05	−0.10	−0.24^*^	0.12	−0.15	0.90^**^	0.67^**^	0.60^**^	–				
11. ACROM-L-T1	7.92	1.74	0.08	0.08	0.05	−0.19	0.19	−0.13	0.72^**^	0.84^**^	0.63^**^	0.76^**^	–			
12. ACROM-L-T2	7.52	1.87	0.14	0.14	0.05	−0.27^*^	0.04	−0.07	0.61^**^	0.69^**^	0.89^**^	0.63^**^	0.72^**^	–		
13. UWC-T0	4.44	2.14	0.02	0.02	0.03	0.08	0.13	0.06	0.09	−0.11	−0.28^**^	0.10	−0.06	−0.25^*^	–	
14. UWC-T1	5.79	2.94	0.10	0.06	0.13	0.23^*^	0.12	0.06	−0.31^**^	−0.27^**^	−0.49^**^	−0.29^**^	−0.21^*^	−0.43^**^	0.58^**^	–
15. UWC-T2	5.27	2.77	0.10	−0.07	0.08	0.26^*^	0.22^*^	0.03	−0.14	−0.17	−0.37^**^	−0.17	−0.19	−0.40^**^	0.52^**^	0.68^**^
16. LWC-T0	4.65	2.72	0.13	0.02	0.05	−0.06	0.09	0.10	0.07	−0.07	−0.14	0.12	−0.03	−0.13	0.66^**^	0.29^**^
17. LWC-T1	6.00	2.88	0.25^*^	0.05	0.18	0.10	0.13	0.04	−0.24^*^	−0.27^**^	−0.37^**^	−0.24^*^	−0.18	−0.33^**^	0.52^**^	0.63^**^
18. LWC-T2	5.37	2.82	0.24^*^	0.10	0.28^**^	−0.08	0.09	−0.04	−0.16	−0.19	−0.34^**^	−0.09	−0.10	−0.31^**^	0.515^**^	0.57^**^
19. F/C-T0	87.49	7.46	−0.01	−0.14	−0.02	−0.27^**^	0.04	−0.09	0.14	−0.02	0.07	0.17	0.05	0.19	0.08	0.03
20. F/C-T1	87.89	8.84	−0.03	−0.09	0.00	−0.13	−0.05	−0.17	0.01	−0.11	0.06	0.08	0.02	0.16	0.00	0.13
21. F/C-T2	85.92	14.04	0.06	−0.06	−0.03	−0.06	−0.03	−0.03	0.02	−0.09	−0.09	0.13	0.06	0.03	0.18	0.11
22. HR-T0	76.70	12.92	−0.14	−0.19	−0.20	−0.33^**^	0.04	−0.06	0.14	0.05	0.08	0.14	0.05	0.10	−0.08	−0.16
23. HR-T1	76.30	11.96	−0.11	−0.11	−0.16	−0.19	0.11	0.00	0.19	0.04	0.07	0.17	0.03	0.10	0.03	−0.09
24. HR-T2	77.74	12.33	−0.19	−0.09	−0.16	−0.27^*^	−0.01	−0.04	0.09	0.07	0.09	0.12	0.03	0.11	−0.02	−0.13
25. SpO2-T0	97.23	1.37	0.10	0.04	0.11	−0.02	−0.13	−0.09	−0.24^*^	−0.15	−0.25^*^	−0.15	−0.12	−0.20	0.03	0.05
26. SpO2-T1	97.26	1.78	0.04	0.12	0.10	0.14	−0.07	0.27^**^	−0.29^**^	−0.20	−0.15	−0.27^**^	−0.23^*^	−0.17	−0.05	0.08
27. SpO2-T2	97.29	2.01	0.15	0.19	0.17	0.03	−0.11	0.01	−0.27^**^	−0.22^*^	−0.22^*^	−0.16	−0.14	−0.16	0.13	0.23^*^

Outcomes	M	SD	15.	16.	17.	18.	19.	20.	21.	22.	23.	24.	25.	26.	27.
01. PPT-T0	3.45	1.50													
02. PPT-T1	3.90	1.80													
03. PPT-T2	4.21	1.77													
04. VAS-T0	2.17	1.78													
05. VAS-T1	1.19	1.41													
06. VAS-T2	1.15	1.45													
07. ACROM-R-T0	8.00	2.21													
08. ACROM-R-T1	8.09	1.88													
09. ACROM-R-T2	7.81	1.92													
10. ACROM-L-T0	7.74	2.07													
11. ACROM-L-T1	7.92	1.74													
12. ACROM-L-T2	7.52	1.87													
13. UCW-T0	4.44	2.14													
14. UCW-T1	5.79	2.94													
15. UCW-T2	5.27	2.77	–												
16. LCW-T0	4.65	2.72	0.30^**^	–											
17. LCW-T1	6.00	2.88	0.53^**^	0.63^**^	–										
18. LCW-T2	5.37	2.82	0.45^**^	0.62^**^	0.76^**^	–									
19. FEV1/CVF-T0	87.49	7.46	0.04	0.00	−0.06	0.06	–								
20. FEV1/CVF-T1	87.89	8.84	−0.04	−0.08	−0.07	0.07	0.47^**^	–							
21. FEV1/CVF-T2	85.92	14.04	0.06	0.09	0.07	0.13	0.25^*^	0.27^**^	–						
22. HR-T0	76.70	12.92	−0.10	−0.01	−0.06	−0.06	0.22^*^	0.27^**^	0.12	–					
23. HR-T1	76.30	11.96	−0.09	0.00	−0.05	0.03	0.35^**^	0.17	0.04	0.60^**^	–				
24. HR-T2	77.74	12.33	−0.24^*^	−0.08	−0.18	−0.11	0.31^**^	0.14	0.13	0.66^**^	0.64^**^	–			
25. SpO2-T0	97.23	1.37	0.10	−0.03	−0.01	0.14	−0.07	0.07	0.06	−0.02	0.01	0.05	–		
26. SpO2-T1	97.26	1.78	0.00	−0.01	−0.01	0.10	−0.18	−0.09	−0.08	0.04	−0.03	0.10	0.53^**^	–	
27. SpO2-T2	97.29	2.01	0.11	0.20	0.24^*^	0.25^*^	−0.03	0.05	0.06	−0.01	0.03	0.01	0.56^**^	0.56^***^	–

^*^p < 0.01; ^**^p < 0.01; T0, baseline; T1, post-test; T2, follow-up. PPT, pain pressure threshold; VAS, visual analog scale; ACROM-R, acromion-bed right; ACROM-L, acromion-bed left; UCW, upper chest wall; LCW, lower chest wall; FEV/CVF, FEV1/FVC ratio (%); HR, heart rate; SpO2, oxygen saturation (SpO_2_).

### Model A

**Figure 2:**
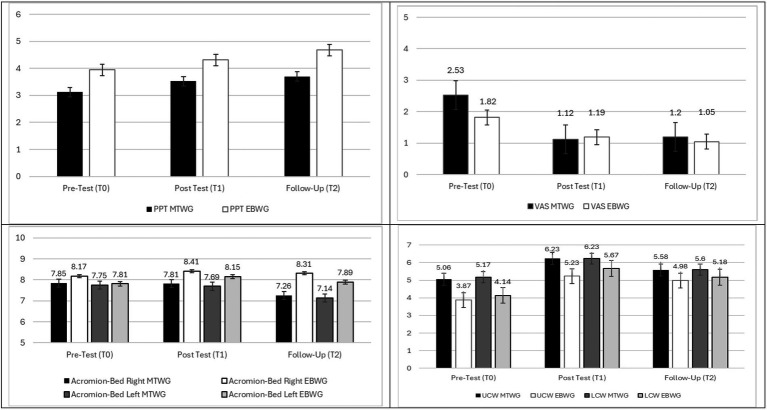
Estimated means for musculoskeletal pain and postural pattern across treatment groups (MTWG and EBWG).

Univariate tests revealed a moderate, statistically significant between-subjects effect on PPT [*F* (1, 85) = 7.03, *p* < 0.01, partial η^2^ = 0.08; Cohen’s *d* = 0.57]. Conversely, no between-subjects effect was detected for VAS [*F* (1, 85) = 1.02, ns]. Table 3 and Figure 2 show that the EBWG reported significantly higher PPT scores than the MTWG across all datapoints. Model A also detected large within-subjects differences for both PPT [*F* (2, 170) = 12.01, *p* < 0.001, partial η^2^ = 0.12; Cohen’s *d* = 0.75] and VAS [*F* (2, 170) = 28.93, *p* < 0.001, partial η^2^ = 0.25; Cohen’s *d* = 1.17] scores. Finally, a statistically significant, between-within-subjects interaction effect was found for VAS scores [*F* (2, 170) = 3.55, *p* < 0.05; partial η^2^ = 0.04; Cohen’s *d* = 0.40], with the EBWG group showing lower baseline levels than the MTWG group at T1, but subsequently stabilizing at T2 and T3. *Post-hoc* tests showed that the change between T2 and T3 was non-significant for PPT (I-J = 0.27, SE = 0.12, ns) nor for VAS (I-J = −0.03, SE = 0.15, ns). These results provide partial support for H1a and H1b.

**Table 3:** Estimated marginal means, SE, and 95% CI for primary and secondary outcomes.

Outcomes	Group	Estimated mean (SE), [95% CI]		
		Pre-test (T0)	Post-test (T1)	Follow-up (T2)
Model A				
Pain pressure threshold (PPT)	MTWG	3.12 (0.24), [2.64, 3.60]	3.52 (0.28), [2.96, 4.09]	3.70 (0.28), [3.15, 4.25]
	*W-S effects:*	*–*	*T0 – T1 = −0.40 (0.21), p < 0.16*	***T0 – T2 = −0.58 (0.21), p < 0.02***
		*–*	*–*	*T1– T2 = −(0.18), p < 1.00*
	EBWG	3.95 (0.22), [3.52, 4.39]	4.31 (0.26), [3.80, 4.82]	4.68 (0.25), [4.18, 5.17]
	*W-S effects:*	*–*	*T0 – T1 = −0.35 (0.19), p < 0.19*	***T0 – T2 = −0.72 (0.19), p < 0.001***
		*–*	*–*	*T1– T2 = −0.37 (0.16), p < 0.10*
	*B-S effects:*	***T0 = −0.83 (0.33), p < 0.01***	***T1 = −0.78 (0.38), p < 0.04***	***T2 = −0.98 (0.37), p < 0.01***
Visual analog scale (VAS)	MTWG	2.53 (0.26), [2.02, 3.05]	1.12 (0.22), [0.69, 1.56]	1.20 (0.23), [0.74, 1.66]
	*W-S effects:*	*–*	***T0 – T1 = 1.41 (0.25), p < 0.001***	***T0 – T2 = 1.33 (0.22), p < 0.001***
		*–*	*–*	*T1– T2 = −0.08 (0.23), p < 1.00*
	EBWG	1.82 (0.23), [1.36, 2.28]	1.19 (0.20), [0.80, 1.59]	1.05 (0.21), [0.64, 1.47]
	*W-S effects:*	*–*	***T0 – T1 = 0.63 (0.23) p < 0.02***	***T0 – T2 = 0.76 (0.20), p < 001***
		*–*	*–*	*T1– T2 = −0.14 (0.20), p < 1.00*
	*B-S effects:*	***T0 = 0.72 (0.35); p < 0.01***	*T1 = −0.07 (0.30), p < 0.88*	*T2 = 0.15 (0.31), p < 0.64*
Model B				
Acromion-bed right (ACROM-R)	MTWG	7.85 (0.34), [7.18, 8.52]	7.81 (0.29), [7.23, 8.39]	7.26 (0.29), [6.69, 7.84]
	*W-S effects:*	*–*	*T0 – T1 = 0.04 (0.22), p < 1.00*	*T0 – T2 = 0.58 (0.25), p < 0.07*
		*–*	*–*	***T1– T2 = 0.55 (0.21), p < 0.03***
	EBWG	8.17 (0.31), [7.56, 8.78]	8.41 (0.27), [7.88, 8.94]	8.31 (0.27), [7.88, 8.84]
	*W-S effects:*	*–*	*T0 – T1 = −0.24 (0.20), p < 0.70*	*T0 – T2 = −0.14 (0.23), p < 1.00*
		*–*	*–*	*T1– T2 = 0.10 (0.19), p < 1.00*
	*B-S effects:*	*T0 = −0.32 (0.45), p < 0.49*	*T1 = −0.60 (0.40), p < 0.13*	***T2 = 1.05 (0.39), p < 0.01***
Acromion-bed left (ACROM-L)	MTWG	7.75 (0.32) [7.12, 8.24]	7.69 (0.28), [7.14, 8.24]	7.14 (0.29), [6.57, 7.71]
	*W-S effects:*	*–*	*T0 – T1 = 0.06 (0.20), p < 1.00*	*T0 – T2 = 0.61 (0.26), p < 0.07*
		*–*	*–*	***T1– T2 = 0.55 (0.21), p < 0.03***
	EBWG	7.81 (0.29), [7.24, 8.38]	8.15 (0.25), [7.65, 8.65]	7.89 (0.26), [7.37, 8.41]
	*W-S effects:*	*–*	*T0 – T1 = −0.34 (0.19), p < 0.22*	*T0 – T2 = −0.08 (0.24), p < 1.00*
		*–*	*–*	*T1– T2 = −0.25 (0.20), p < 0.60*
	*B-S effects:*	*T0 = −0.06 (0.43), p < 0.89*	*T1 = −0.46 (0.37), p < 0.22*	*T2 = 0.75 (0.39), p < 0.06*
Model C				
Upper chest wall (UCW)	MTWG	5.06 (0.29), [4.49, 5.53]	6.23 (0.42), [5.40, 7.05]	5.58 (0.42), [4.75, 6.41]
	*W-S effects:*	*–*	***T0 – T1 = −1.17 (0.37), p < 0.001***	*T0 – T2 = −0.52 (0.37), p < 0.48*
		*–*	*–*	*T1– T2 = −0.65 (0.34), p < 0.17*
	EBWG	3.87 (0.28), [3.32, 4.42]	5.23 (0.40), [4.44, 6.03]	4.98 (0.40), [4.18, 5.78]
	*W-S effects:*	*–*	***T0 – T1 = −1.37 (0.36), p < 0.001***	***T0 – T2 = 1.37 (0.36), p < 0.001***
		*–*	*–*	*T1– T2 = −0.25 (0.33), p < 1.00*
	*B-S effects:*	***T0 = 1.19 (0.40), p < 0.004***	*T1 = 0.99 (0.58), p < 0.09*	*T2 = 0.59 (0.58), p < 0.31*
Lower chest wall (LCW)	MTWG	5.17 (0.40), [4.37, 5.97]	6.23 (0.42), [5.40, 7.06]	5.60 (0.42), [4.77, 6.42]
	*W-S effects:*	*–*	*T0 – T1 = −1.06 (0.36), p < 0.01*	*T0 – T2 = −0.43 (0.36), p < 0.71*
		*–*	*–*	*T1– T2 = 0.64 (0.29), p < 0.08*
	EBWG	4.14 (0.39), [3.37, 4.92]	5.67 (0.40), [4.86, 6.47]	5.18 (0.40), [4.38, 5.98]
	*W-S effects:*	*–*	***T0 – T1 = −1.53 (0.35), p < 0.001***	***T0 – T2 = −1.04 (0.35), p < 0.01***
		*–*	*–*	*T1– T2 = 0.48 (0.28), p < 0.25*
	*B-S effects:*	*T0 = 1.03 (0.56), p < 0.07*	*T1 = 0.57 (0.58), p < 0.33*	*T2 = 0.41 (0.58), p < 0.33*
Model D				
Pulmonary function	MTWG	87.69 (1.15), [85.40, 89.97]	88.41 (1.39), [85.66, 91.17]	85.88 (2.18), [81.55, 90.21]
	*W-S effects:*	*–*	*T0 – T1 = −0.73 (1.35), p < 1.00*	*T0 – T2 = 1.80 (2.19), p < 1.00*
		*–*	*–*	*T1– T2 = 2.53 (2.24), p < 0.79*
	EBWG	86.76 (1.08), [84.62, 88.90]	87.17 (1.30), [84.60, 88.90]	85.95 (2.04), [81.90, 90.03]
	*W-S effects:*	*–*	*T0 – T1 = −0*.41 (1.27), p < 1.00	*T0 – T2 = 0*.81 (2.05), p < 1.00
		*–*	*–*	*T1– T2 = −*1.22 (2.10), p < 1.00
	*B-S effects:*	*T0 = 0.93 (1.57), p < 0.56*	*T1 = 1.24 (1.89), p < 0.52*	*T2 = −0.07 (2.98), p < 0.98*
Model E				
Heart rate	MTWG	76.57 (2.06), [72.47, 80.67]	75.62 (1.86), [71.83, 79.22]	78.62 (1.91), [74.83, 82.41]
	*W-S effects:*	*–*	*T0 – T1 = 1.05 (1.76), p < 1.00*	*T0 – T2 = −2.05 (1.65) p < 0.65*
		*–*	*–*	*T1– T2 = −3.10 (1.58), p < 0.16*
	EBWG	76.83 (1.93), [73.00, 80.67]	78.10 (1.74), [74.65, 81.56]	76.98 (1.79), [73.42, 80.53]
	*W-S effects:*	*–*	*T0 – T1 = −1.27 (1.65), p < 1.00*	*T0 – T2 = 0.15 (1.54) p < 1.00*
		*–*	*–*	*T1– T2 = −1.13 (1.47), p < 1.00*
	*B-S effects:*	*T0 = −0.26 (2.83), p < 0.93*	*T1 = −2.58 (2.54), p < 0.31*	*T2 = 1.64 (2.61), p < 0.53*
Oxygen saturation	MTWG	97.26 (0.21), [96.84, 97.69]	96.91 (0.28), [96.36, 97.45]	97.14 (0.31), [96.52, 97.76]
	*W-S effects:*	*–*	*T0 – T1 = 0.36 (0.24), p < 0.42*	*T0 – T2 = 0.12 (0.26), p < 1.00*
		*–*	*–*	*T1– T2 = 0.24 (0.29), p < 1.00*
	EBWG	97.29 (0.20), [96.90, 97.69]	97.56 (0.26), [97.05, 98.08]	97.42 (0.29), [96.84, 98.00]
	*W-S effects:*	*–*	*T0 – T1 = −0.27 (0.23), p < 1.00*	*T0 – T2 = −0.13 (0.25), p < 1.00*
		*–*	*–*	*T1– T2 = 0.15 (0.26), p < 1.00*
	*B-S effects:*	*T0 = −0.03 (0.29), p < 0.92*	*T1 = 0.66 (0.38), p < 0.09*	*T2 = −0.27 (0.43), p < 0.52*

B-S effects, between subjects effect; W-S effects, within subjects effect.

### Model B

No significant between-subjects differences were observed for changes in thorax position. More precisely, estimated mean differences among groups in the left acromion-to-bed distance measure [*F* (1, 88) = 1.42, ns] were not statistically significant. On the other hand, estimated mean differences in the right acromion-to-bed measurement were marginally significant [*F* (1, 88) = 3.02, *p* < 0.10, partial η^2^ = 0.03, Cohen’s *d* = 0.37]. However, Model B detected statistically significant within-subjects differences for the left acromion-to-bed distance scores [*F* (1, 184.80) = 3.93, *p* < 0.05; partial η^2^ = 0.04; Cohen’s *d* = 0.40], but not for right acromion-to-bed scores [*F* (2, 176) = 2.39, ns]. Between-within mean differences among groups were only marginally significant, either for left acromion-to-bed distance [*F* (1, 87, 184.80) = 2.86, *p* < 0.10; partial η^2^ = 0.03; Cohen’s *d* = 0.34] and for right acromion-to-bed [*F* (2, 176) = 2.68, *p* < 0.10; partial η^2^ = 0.03; Cohen’s *d* = 0.34]. These results partially support H2a and H2b.

### Model C

Model C revealed significant between-subjects differences for the upper chest wall [*F* (1, 89) = 4.39, *p* < 0.05, partial η^2^ = 0.02; Cohen’s *d* = 0.28], but not for the lower chest wall [*F* (1, 89) = 1.75, ns]. However, the results of the univariate test revealed significant within-subjects for both the upper [*F* (2, 178) = 13.33, *p* < 0.001, partial η^2^ = 0.13; Cohen’s *d* = 0.77] and lower chest wall scores [*F* (1.85, 169.75) = 15.33, *p* < 0.001, partial η^2^ = 0.15; Cohen’s *d* 0.83]. No between-within subjects effects were found.

Finally, neither Model D nor E revealed statistically significant mean differences across groups for pulmonary function scores (Model D) nor in heart rate (HR) and oxygen saturation scores (Model E). These results do not support H3a or H3b.

## Discussion

**Figure 3:**
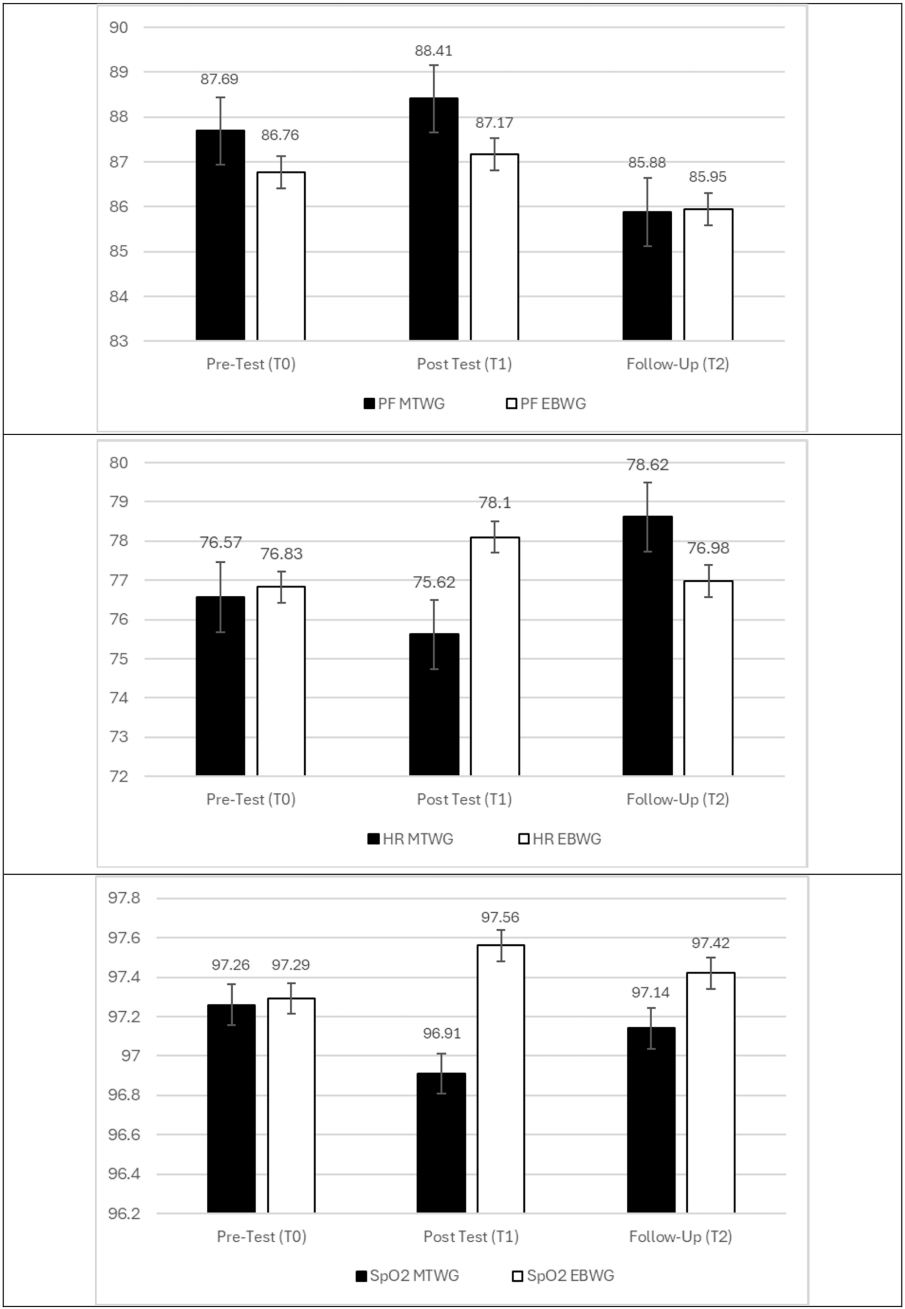
Estimated means for cardiorespiratory outcomes across treatment groups (MTWG and EBWG).

Our findings revealed interesting results across the different variables studied. Regarding pain, both treatments showed positive outcomes, confirming the initial hypothesis that both interventions are beneficial in reducing perceived pain and its intensity. In terms of postural pattern, assessed by the acromion-to-bed distance, results were more favorable for the MTWG, whereas upper thoracic expansion was more effectively improved through the EBWG intervention. Finally, for lower thoracic expansion, a trend toward improvement was observed in both groups, although without statistically significant results. No changes were found in cardiorespiratory parameters, as participants were in optimal conditions from the beginning and throughout the study (Figure 3).

In relation to pain, both groups showed an improvement throughout the assessments for the two pain variables. Considering pain is a prevalent condition in older adults, our results show two treatment options which are sustainable, simple, and affordable, such as manual therapy and resistance bands, both combined with high-intensity walking. In this regard, in relation to the two pain variables, the MTWG showed poorer scores than the EBWG in both of them in the first assessment, and this trend was maintained throughout the study. For trapezius PPT, our results showed statistical differences between groups in favor of the EBWG. This may be due to the fact that elastic band exercises help to build strength, improve flexibility, and enhance overall fitness, and when applied as a moderate-intensity resistance training it is an optimal rehabilitation option for older adults (56). As suggested by other authors, programmes that achieve positive results in pain relief for older adults are those that combine interventions such as Pilates and basic strength training (57). Our study combined resistance elastic band exercises with aerobic exercise such as high-intensity walking. This could have enhanced the effect of elastic band use since combining resistance and aerobic exercises has shown to improve mobility, muscle quality and strength in older adults (24, 26). Our study innovates by highlighting the improvements in terms of musculoskeletal pain. Although previous research had also shown a reduction in pain when combining walking with resistance elastic band training (58), it focused on young adults (see Figure 2).

The improvements achieved in the EBWG compared to the MTWG were mainly related to the PPT. This might be explained by the fact that two of the elastic band exercises activated the shoulder girdle focusing on upper and lower trapezius muscles stabilizing the scapula, retracting the shoulder blades, and participating in the rotation and lateral flexion of the neck. Therefore local muscle endurance training could be beneficial, due to improved blood circulation, when there is poor capillarisation of trapezius muscle fibers (59), something typical of the aging process (26). The MTWG also included techniques targeting the trapezius muscle, but the intervention was passive and focused less on this area, applying only trigger point inhibition in the trapezius followed by its subsequent stretching. This might have a lesser effect compared to strength training. In other words, the treatment was more focused on muscle release rather than endurance.

Moreover, the superior effects observed in the EBWG can be further explained from the perspective of pain physiology. Resistance training regulates the release of neurotransmitters and signaling proteins involved in nociceptive pathways, including *β*-endorphins, substance P, anti-inflammatory cytokines, and endogenous endocannabinoids. *β*-endorphins and endocannabinoids contribute to endogenous analgesia, while the inhibition of substance P reduces the transmission of nociceptive signals to the thalamus. Peripherally, the release of anti-inflammatory cytokines counteracts proinflammatory mediators. Furthermore, elastic band exercises activate the shoulder girdle and enhance local muscle endurance, which may lead to the desensitization of hyperactive muscle spindles and normalization of muscle tone. The repeated activation of large-diameter afferent fibers (A-beta) also competes with nociceptive signals from A-delta and C fibers, in line with the Gate Control Theory, further reducing pain perception. Altogether, these integrated physiological mechanisms provide a plausible explanation for the greater benefits observed in the EBWG, linking the intervention to both peripheral and central changes in pain processing (60).

Regarding pain measured on the VAS, both groups showed within-subject improvements in this self-reported variable, suggesting that both approaches are effective for relieving non-specific back pain. Pain is a subjective, complex, and multidimensional experience for which no objective biological markers currently exist.

Regarding pain measured on VAS, both groups showed within-subjects differences in this self-reported variable, so both approaches appear to be optimal for relieving non-specific back pain. Pain is a subjective, complex and multidimensional experience for which there are no objective biological markers; despite decades of effort, there is no neurophysiological or chemical test that can measure pain in individual patients. Self-reporting is considered the most accurate and appropriate pain assessment method as health care professionals often underestimate a patient’s pain (5). However, our results offer some hope of offering a relief option for older adults suffering from back pain, even if they are without a specific diagnosis explaining the cause of pain. This pain undoubtedly has a negative impact on their quality of life (61).

Regarding postural pattern, there is evidence that supports that body mechanics in older adults are diminished due to regressive changes in ligaments and articular cartilage (62), especially in women. In addition, musculoskeletal changes occur in the thorax, such as osteoarticular stiffness of the rib cage, dehydration of intervertebral discs, and progressive ossification of chondrocostal and costovertebral joints. All these postural changes frequently accompany the aging process. In our study, the EBWG showed no improvements compared to the MTWG in relation to changes in acromion-to-bed distance. Manual therapy techniques managed to reduce the acromion-to-bed distance from T1 to T2, thus, shoulders exhibited less anterior displacement. This result can be explained by the inclusion of two techniques in the manual therapy (MT) protocol specifically aimed at improving clavicular mobility (gliding of the sternoclavicular joint) and scapular mobility (scapulothoracic joint mobilization and sliding of the thoracic vertebral joints). These techniques involve movement of the shoulders and clavicle, and particularly emphasize scapular mobility, which engages the thoracic vertebrae and the scapular stabilizing musculature. It is possible that the protraction and retraction of the scapula involving the trapezius, rhomboids, serratus anterior, and pectoralis minor muscles, whose function is to stabilize the scapula (63), are key to this improvement. Additionally, the contribution of clavicular mobility and its role in the scapulohumeral rhythm (64) may further explain these benefits.

On the other hand, with regard to upper thoracic mobility, both treatments achieved improvements at the end of the intervention. However, EBWG results were more sustained compared to the MTWG, likely because the exercises were specifically focused on thoracic expansion (trunk extensor exercise). This improvement has also been confirmed by other authors (65), although this had not previously been demonstrated using resistance band training. Elastic band exercises for the thoracic region may be more engaging than traditional mobility exercises. This is because they add variety, allow for adjustable resistance—albeit subjectively—are portable, safe, and provide visual motivation by allowing participants to see and feel the band stretching. Moreover, resistance training with elastic bands has been shown to have a positive impact on both physical and mental health (66).

In terms of lower thoracic mobility/expansion, both protocols showed a trend toward improvement, but the results were not statistically significant. These findings are consistent with the nature of the exercises and interventions applied, which were not specifically targeted at the lower thoracic region. On the one hand, the elastic band exercises did not promote focused expansion of the lower thoracic area, and the self-assisted manual therapy protocol primarily addressed general mobility. Therefore, more targeted interventions might have yielded better outcomes.

Regarding respiratory variables (FEV1/FVC), the pattern observed in our sample indicates neither an obstructive nor a restrictive profile. This suggests that we are working with relatively healthy older adults, whose values fall within normative ranges. Similarly, heart rate (HR) and oxygen saturation (SpO₂) remained within normal limits throughout the study. The chest mobilization technique has been used in clinical practice to enhance thoracic cage mobility and modulate the autonomic nervous system through the activation of proximal ganglia of the thoracic sympathetic chain at the costotransverse joint. In this regard, Rocha et al. conducted a pilot study evaluating the feasibility of an intervention protocol and the immediate effects of two manual rib cage lift techniques on the autonomic nervous system in patients with Chronic Obstructive Pulmonary Disease. The study concluded that diaphragmatic release may reduce resting heart rate and increase heart rate variability following the intervention (67). However, our sample consisted of individuals without known respiratory or cardiac conditions, who are also health-conscious, which may explain their optimal baseline status in these variables. Therefore, it can be concluded that while both intervention protocols were effective in improving parameters related to pain, mobility, and posture, neither was able to induce significant changes in respiratory or cardiovascular variables.

Our results indicate that this is a population that actively takes care of their health. Other authors have highlighted that inadequate health literacy is associated with poorer physical and mental health outcomes (68). In conclusion, we are observing a generation of older adults who are increasingly aware of the importance of physical exercise, and this factor positively influences their respiratory values, which were found to be satisfactory in this population.

Social, economic and biomedical factors are in continuous change, and the increased life expectancy of the population has an impact on the health system with a considerable increase in expenditure for social security and assistance (69). In order to avoid this situation, an active and healthy aging process necessary. Given today’s long-lived society, it is essential to achieve longer and healthier lives, in addition to reducing the costs of this type of society (70). According to research by Barrios Duarte et al. (71) exercise in older adults will provide multiple benefits such as decreased pain, improved quality of life, improved mood or improved sleep. It would be desirable to implement home-based follow-up using technological tools or monitoring through primary care centers, in order to enhance motivation and ensure proper supervision.

### Limitations and strengths

Our study is not without limitations. First, there were no exercises or techniques specifically targeting the lower thoracic region, which may have limited the outcomes in that area. In future studies, it would be advisable to incorporate techniques into both protocols—self-assisted manual therapy and elastic band mobility exercises—with a focus on diaphragmatic breathing. Moreover, the postural assessment used (acromion-to-bed distance), while practical, may lack the sensitivity of more precise methods such as digital inclinometry or photogrammetry. Additionally, the sample was restricted to community-dwelling older adults (60–80 years), excluding frail or institutionalized individuals, which may limit the generalizability of our findings. Finally, including a control group could provide more robust comparisons between the two intervention protocols.

The strength of the present study lies in the fact that both protocols can be initiated and implemented at home, empowering individuals to take an active role in managing and improving their health. Moreover, the use of validated assessment tools (PPT, VAS, acromion-to-bed test, chest expansion, and spirometry) strengthens the reliability of the outcome measures.

## Conclusion

Both protocols relieve pain; however, the manual therapy intervention alleviates nonspecific back pain, while the elastic band intervention improves the musculoskeletal pain pressure threshold in the trapezius muscles. The postural pattern related to the acromion-to-table distance improves with manual therapy treatment, whereas upper thoracic expansion improves with elastic band treatment. Neither protocol succeeded in improving lower thoracic mobility parameters or respiratory variables.

## Data Availability

The raw data supporting the conclusions of this article will be made available by the authors, without undue reservation.

## Footnotes

^1^ https://www.herycor.com/#
