# Supplementary material for: Comparative effectiveness of manual therapy and band exercises combined with high-intensity walking for pain, posture, and cardiorespiratory health in older adults: a randomised clinical trial
Source: Front Med (Lausanne). 2025 Oct 29;12:1654670. doi: 10.3389/fmed.2025.1654670 (PMC12605172; doi:10.3389/fmed.2025.1654670)
Supplement: Supplementary file 3 [file Table_3.DOCX]

**APPENDIX II. ANALYTICAL STRATEGY AND ROBUSTNESS CHECKS.**

**DATA ANALYSIS**

General linear models, such as an RM-MANOVA, have substantive advantages over linear regression models when testing the efficacy of interventions. For example, an RM-MANOVA approach can correct the effect of correlation between dependent variables and time effects (auto-correlations). Moreover, the RM-MANOVA approach allows for testing the statistical significance of between-subject and within-subject effects, as well as their possible interaction effect (between-within interaction effect). Further, when the assumptions for a general linear model are met, corrected means for each group can be easily derived from the general linear model (i.e., estimated marginal means, or EMM). In the present study, EMMs for each group were calculated, and Bonferroni's correction was applied when conducting pairwise comparisons.

In the present study, five RM-MANOVA models were generated (Models A, B, C, D and E). For our between-subjects factor, we entered a dummy coded variable, in which the walking + MT (W+MT) group was coded as "0," and the walking + EB (W+EB) group was coded as "1." For our within-subject variables, three data points were (1) baseline, (2) post-intervention, and (3) follow-up (one month after) were entered. No control variables were included in any of the five linear models.

For Model A, the dependent variables were aggregated scores of PPT and VAS, with one value for each of the three temporal data points measured (T0, T1, and T2, respectively). In Model B, APOSR and APOSL were used as dependent variables. Again, each metric was assessed at the same three temporal data points as specified in Model A: (1) baseline, (2) post-intervention, and (3) follow-up. UCW and LCW were used as dependent variables in Model C. The FEV1/FVC ratio (%) for each data point was entered in Model D as dependent variables. Finally, Heart rate (HR) and oxygen saturation (SaO2) for all data points were entered in Model E as dependent variables.

Box's M and Mauchly's Sphericity tests were conducted to ensure that our RM-MANOVA met the assumptions of a general linear model. Again, ensuring these assumptions are met is important to ensure that the estimated marginal means are trustworthy. More precisely, Box's M is a statistic that tests the null hypothesis that the covariance matrices in the multivariate solution are equal across groups (i.e., the homogeneity of variance assumption). A non-significant p-value would indicate that this assumption has been met.

Mauchly's Test of Sphericity follows the same logic for the within-subject component of an RM-MANOVA. Again, a non-significant p-value for this test would indicate that the sphericity assumption has been met. However, if the Sphericity test fails, certain corrections can be applied based on the level of the Epsilon statistic. More precisely, the Greenhouse-Geisser correction should be applied if epsilon < .75. Instead, if epsilon > .75, the Huynh-Felt correction should be applied to the degrees of freedom in subsequent univariate F-tests.

The results of an RM-MANOVA can be interpreted as follows. First, the trustworthiness of the overall model and its derived parameters must be determined using the tests as the ones mentioned above (Box's M and Mauchly's W). If the multivariate solution is deemed untrustworthy, then univariate tests should be preferred (Hair et al., 2006). When interpreting the results of a multivariate solution, if the p-value of the Wilks' Lamba statistic for a given predictor is statistically significant at p < .05 in the multivariate solution, that would mean that said predictor is associated with all dependent variables. For the univariate solution, Fischer’s Test (F-Test) is used to test for statistical significance.

If the Sphericity assumption is not met for the univariate solution, the F-test can be corrected employing the Greenhouse-Geisser or the Huynh-Feldt corrections. More precisely, these corrections adjust the degrees of freedom used to calculate the F statistic. If the significant test of the (corrected) F-value is significant at the p < .05 level, that result would support an association between the predictor (e.g., treatment) and each dependent variable.

Lastly, an RM-MANOVA analysis reports Partial Eta squared (partial η^2^) as an indicator of effect size rather than Cohen's d. Therefore, to ease the interpretation of the effect size, any partial η^2^ scores must be transformed into Cohen's d effect sizes to ease its interpretation. According to Cohen's method, the magnitude of the effect is classified as small (0.20–0.49), moderate (0.50–0.79), or large (> 0.80) [19].

**ROBUSTNESS CHECKS**

**Correlations between outcome measures**

**Model A.** Table XX1 shows that, as we expected, PPT-T0 was significantly and strongly correlated with PPT-T1 (r = .68, p <.001) and PPT-T2 (r = .68, p <.001). In turn, PPT-T1 was strongly correlated with PPT-T2 (r = .72, p <.001). Similarly, VAS-T0 was significantly and moderately correlated with VAS-T1 (r = .34, p <.001) and strongly correlated with VAS-T2 (r = .47, p <.001). Further, VAS-T1 was correlated with VAS-T2 (r = .58, p <.001).

**Model B.** Table XX1 shows that APOSR-T0 was significantly correlated with APOSR-T1 (r = .80, p < .001) and APOSR-T2 (r = .67, p< .001). Similarly, APOSR-T1 was significantly correlated to APOSR-T2 (r = .73, p< .01). Also as expected, APOSL-T0 was significantly correlated with APOSL-T1 (r = .77, p < .001) and APOSL-T2 (r = .63, p< .001). Similarly, APOSL-T1 was significantly correlated to APOSL-T2 (r = .72, p< .01). When taken as a whole, these correlations justify the use of an RM-MANOVA approach.

**Model C.** Table XX1 shows that UCW-T0 was significantly correlated with UCW-T1 (r = .61, p < .001) and UWC-T2 (r = .53, p< .001). Similarly, UCW-T1 was significantly correlated to UCW-T2 (r = .74, p< .001). Also as expected, LCW-T0 was significantly correlated with LCW-T1 (r = .62, p < .001) and LCW-T2 (r = .59, p< .001). Similarly, ULW-T1 was significantly correlated to LCW-T2 (r = .76, p< .01). When taken as a whole, these correlations justify the use of an RM-MANOVA approach.

**Model D.** Table XX1 shows that FEV1/FVC-T0 was significantly correlated with FEV1/FVC-T1 (r = .73, p < .001) and FEV1/FVC-T2 (r = .75, p< .001). Similarly, FEV1/FVC-T1 was significantly correlated to FEV1/FVC-T2 (r = .73, p< .01). When taken as a whole, these correlations justify the use of an RM-MANOVA approach.

**Model E.** Finally, Table XX1 shows that FC-T0 was significantly correlated with FC-T1 (r = .57, p < .001) and FC-T2 (r = .62, p< .001). Similarly, FC-T1 was significantly correlated to FC-T2 (r = .65, p< .01). Also as expected, SAO2-T0 was significantly correlated with SAO2-T1 (r = .36, p < .001) and APOSL-T2 (r = .42, p< .001). Similarly, SAO2-T1 was significantly correlated to SAO2-T2 (r = .51, p< .001). When taken as a whole, these correlations justify the use of an RM-MANOVA approach.

**ASSUMPTIONS CHECK - GENERAL LINEAR MODELS**

**Model A.** Box's M test for Model A was significant (M = 47.17; F (21, 24258.82) = 2.07, p <.01), suggesting that the homogeneity of variance assumption is not met, and thus the multivariate solution is untrustworthy. Hence, univariate tests should be preferred. However, Mauchly's Sphericity test was non-significant for neither PPT (W _(2)_ = .97; χ2 _(2)_ = 2.27; p < .32) nor for VAS (W _(2)_ = .97; χ2 _(2)_ = 3.04; p < .22). This result indicates that the sphericity assumption was met. Therefore, sphericity can be assumed when interpreting the F-test in the univariate analyses.

**Model B.** Box's M test for Model B was significant (M = 37.61; F (21 26619.32) = 1.66, p <.03), suggesting, again, that the multivariate solution should be deemed untrustworthy. Mauchly's Sphericity test was non-significant for acromion and bed – right (W _(2)_ = .94; χ2 _(2)_ = 5.43; p < .07), but significant for acromion and bed – left (W _(2)_ = .90; χ2 _(2)_ = 9.45; p < .01). Moreover, the Epsilon statistic was ε = .94 for acromion and bed – left. This means that sphericity can be assumed for acromion and bed – right when interpreting the univariate F-test (no correction needed), but for acromion and bed – left the Huynh-Feldt correction should be applied to F-test’s degrees of freedom.

**Model C.** A detailed inspection of the results of Box's M (M = 63.59; F (21, 28,860.03) = 2.81, p <.001) revealed that the multivariate solution was untrustworthy. Hence, the univariate solution should be preferred. As it occurred for Model B, Mauchly's Sphericity test was non-significant for UWC scores (W _(2)_ = .99; χ2 _(2)_ = 1.13; p < .57), but significant for LWC scores (W _(2)_ = .92; χ2 _(2)_ = 7.52; p < .05). Furthermore, because the epsilon statistic was ε = .92, all degrees of freedom employed in the F-tests comparing means for LWC must corrected using the Huynh-Feldt correction.

**Model D.** A detailed inspection of the results of Box's M test for Model D revealed that the multivariate solution was not trustworthy (M = 19.24; F (6, 53425,04) = 3.09, p <.01). Similarly, Mauchly's W statistic was significant (W _(2)_ = .72; χ2 _(2)_ = 28.62; p < .001), with an epsilon value of ε = .80, indicating that the Huynh-Feldt correction is required to interpret the F-test for within-subject differences in this model.

**Model E.** As it occurred for Model D, Model E did not pass Box's M test (M = 44.63; F (21, 27409.83) = 1.97, p <.001) indicating that the homogeneity of variance assumption was not met, and a univariate solution should be preferred. However, Mauchly's W statistic was non-significant for FC (W _(2)_ = .98; χ2 _(2)_ = 1.47; p < .48) and SAO2 (W _(2)_ = .97; χ2 _(2)_ = 2.59; p < .27) suggesting that sphericity can be assumed when interpreting the F-test when testing for statistical differences within-subjects and in any between-within subject interaction.
