# Supplementary material for: Comparative effectiveness of manual therapy and band exercises combined with high-intensity walking for pain, posture, and cardiorespiratory health in older adults: a randomised clinical trial
Source: Front Med (Lausanne). 2025 Oct 29;12:1654670. doi: 10.3389/fmed.2025.1654670 (PMC12605172; doi:10.3389/fmed.2025.1654670)
Supplement: Supplementary file 4 [file Table_4.DOCX]

APPENDIX III

| **SELF-ASSISTED MANUAL THERAPY PROTOCOL** | | | |
| --- | --- | --- | --- |
| **Manual Therapy Tecniques** | **Explanation** | **Image** | **Time** |
| **1. Practice breathing: with abdominal area and**  **thoracic area.** | **Position:** lying on your back (if you have back discomfort you can bend your legs).  **Perform**: 5 breaths directing the air towards the abdominal area, another 5 towards the upper chest area and 5 combining both areas. | 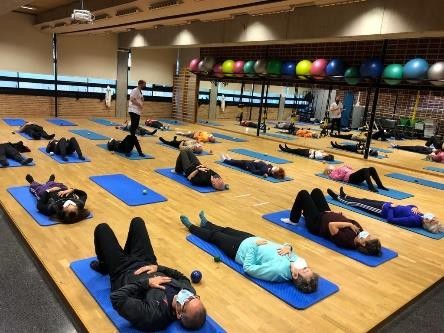 | 1 minute |
| **2. Isometric with suboccipital flexion.** | **Position:** lying on your back (if you have back discomfort you can bend your legs) and with a soft ball of small diameter placed on the occipital bone of the head.  **Perform:** breathe in in the starting position, on the exhale bring the chin slightly towards the chest (suboccipital flexion) and maintain the position by lightly pressing the ball for a few seconds. | 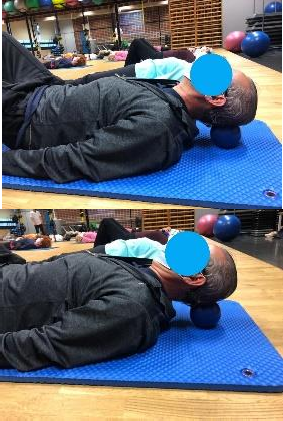 | 3 minutes |
| **3. Fast circular rubbing (neuro-**  **lymphatic).** | **Position:** Standing with index and middle fingers of both hands around the sternum and practicing mindful breathing.  **Perform:** quick gentle rubbing with the fingers for 30 seconds on 2 points in the anterior area (below the clavicle and mammillary line) and 2 in the posterior area that are performed with the knuckles (last ribs and final lumbar area). | 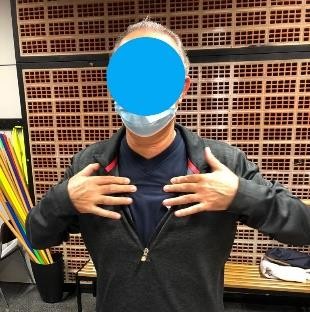 | 4 minutes |

| **4. Move your head forward and backward.** | **Position:** standing, with the body well positioned and practicing conscious breathing.  **Perform**: bring the head forward during exhalation (stretching the suboccipital) and then backward during inspiration. | 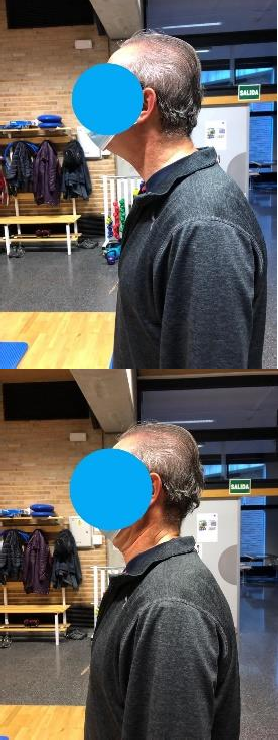 | 3 minutes |
| --- | --- | --- | --- |
| **5. Dotting and stretching the sternocleidomastoid**  **(ECOM).** | **Position:** standing, with the body well positioned and practicing conscious breathing. With the hands in a pincer, both ECOM muscles are located.  **Perform:** with your pincer hands, the ECOM muscles on both sides are stippled at the same time. Next, the muscle is stretched on one side: with the help of the hands, the ends of the muscle are stretched while the head is turned to the opposite side. It is repeated on the other side. | 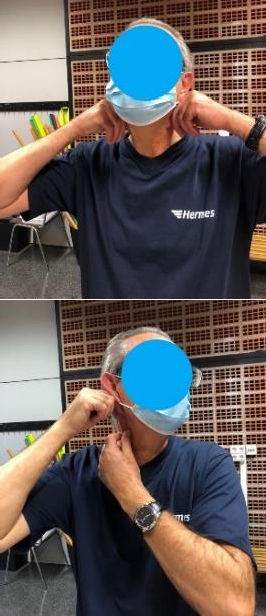 | 4 minutes |

| **6. Inhibition and stretching of the**  **trapeze.** | **Position:** Patient standing, bring the hand to the opposite trapezius (area between the shoulder and the cervical spine).  **Perform**: The hand on the trapezius applies a sustained pressure for 90 seconds. After this time, with the same hand that has applied the pressure, he now puts it on the head and forces the tilt of the head a little to be able to perform the stretch of the trapezius that has been pressed.  It is repeated with the trapeze on the other side. | 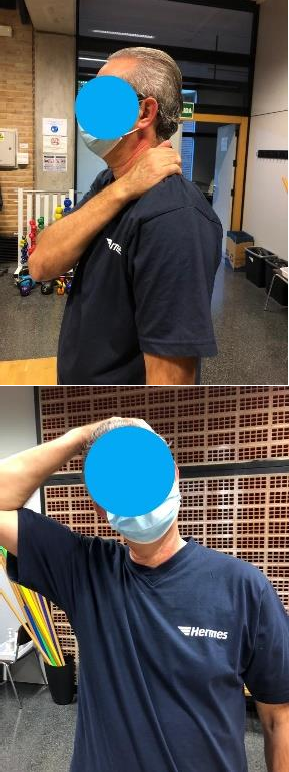 | 4 minutes |
| --- | --- | --- | --- |
| **7. Anterior and posterior thoracic expansion.** | **Position**: standing, place your hands on the last ribs to accompany the opening and closing of the thoracic.  **Perform**: In inspiration we accompany in the opening and in the exhalation we accompany in the closing.  It is done two minutes at the front and two at the back. | 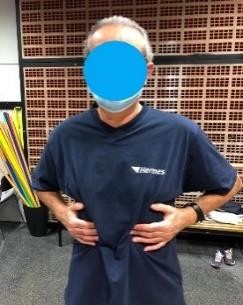 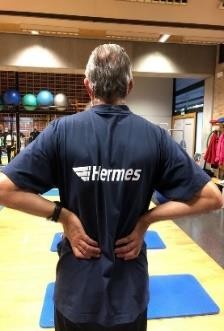 | 4 minutes |

| **8. Skin fold at the last rib.** | **Position**: standing, with the body well positioned and practicing conscious breathing.  **Perform:** take the fold of skin that is under the last rib and holding it in place take several breaths.  Then it is released little by little. | 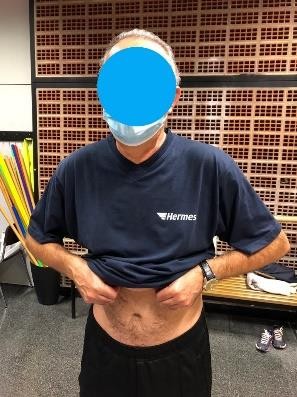 | 3 minutes |
| --- | --- | --- | --- |
| **9.Scapular protraction.** | **Position**: standing and with arms stretched out in front of you.  **Perform**: during inspiration try to bring the scapulae together and in exhalation separate them. | 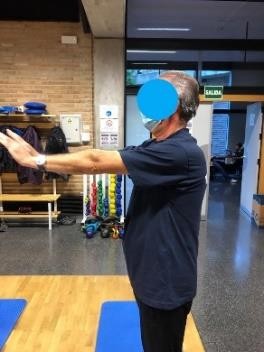 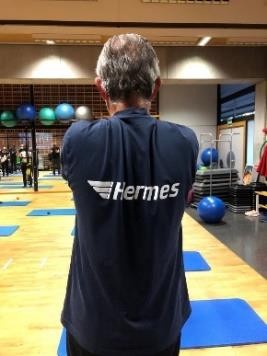 | 3 minutes |
| **10. Practice Breathing: With Zone**  **abdominal and thoracic area.** | **Position:** lying on your back (if you have back discomfort you can bend your legs).  **Perform**: 5 breaths directing the air towards the abdominal area, another 5 towards the upper chest area and 5 combining both areas. | 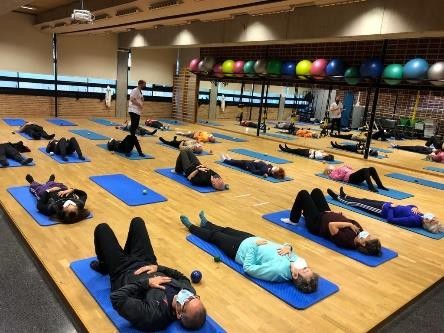 | 1 minute |
